# Association between types of social isolation and all-cause mortality among the survivors in the disaster-affected area of the great East Japan earthquake: The RIAS study

**DOI:** 10.1016/j.pmedr.2026.103654

**Published:** 2026-09-30

**Authors:** Satoru Kanda, Eri Takusari, Haruki Shimoda, Kiyomi Sakata, Kozo Tanno, on behalf of the RIAS Study Group

**Affiliations:** a Department of Hygiene and Preventive Medicine, School of Medicine, Iwate Medical University, Iwate, Japan; b Iwate Prefectural Government, Morioka, Iwate, Japan; c Department of General Medicine, School of Medicine, Iwate Medical University, Iwate, Japan

**Keywords:** Great East Japan Earthquake, Social isolation, Disaster, Mortality, Cohort study

## Abstract

**Objective:**

We aimed to determine whether different types of social isolation after a disaster are associated with all-cause mortality, which currently remains unknown, using data from a prospective cohort study of survivors in an area affected by the Great East Japan Earthquake.

**Methods:**

In total, 9311 participants (3600 men and 5711 women) were followed up from October 1, 2011, to the occurrence of censoring events: death, loss to follow-up, or the end of the follow-up period (December 31, 2017). Social isolation types were assessed using the Lubben Social Network Scale-6 (LSNS-6) Family Subscale and LSNS-6 Friend Subscale (using 5/6 as a cutoff point). Using Cox proportional hazards models, the hazard ratios of all-cause mortality according to the social isolation type (no isolation as the reference) were estimated.

**Results:**

Social isolation from friends, but not from family members, was significantly associated with an increased risk of all-cause mortality (hazard ratio, 1.28; 95% confidence interval, 1.10,1.63).

**Conclusions:**

Social isolation from friends contributes to an increased risk of all-cause mortality. More focused preventive healthcare and medical support for premature death after a disaster and the preservation of social networks are required for disaster survivors who are socially isolated from friends.

## Highlights

• We examined if different types of social isolation after disasters are associated with all-cause mortality.
• No significant differences were observed in comprehensive and family members' social isolation.
• Social isolation from friends increases the risk of premature death after a large-scale disaster.

## 1 Introduction

In recent times, >10,000 people in Japan have been affected by natural disasters annually (https://www.stat.go.jp/data/nenkan/74nenkan/29.html, n.d.). Experiencing a disaster has various adverse effects on both physical and mental health (Hong and Efferth, 2016; Jang et al., 2021; Adu et al., 2023), posing a significant challenge for public health. A recent meta-analysis of 90 prospective cohort studies showed that individuals with social isolation had a 1.3 times higher risk of all-cause mortality in the general population (Wang et al., 2023). A previous study showed that social isolation was significantly associated with an increased risk of all-cause mortality with a 1.38-fold higher hazard ratio in men and 1.49-fold higher hazard ratio in women in the area of the Great East Japan Earthquake (GEJE) (Kotozaki et al., 2022). The adverse effects of social isolation on health can increase even in disaster areas, with the loss of help caused by a lack of connection with others.

Social isolation is generally assessed as a construct encompassing relationships and multiple social ties, such as family members and friends. Few studies have examined the association between isolation, based on the type of social tie, and mortality (Wang et al., 2023; Lubben et al., 2006). In the general population in a non-disaster setting, social isolation from family members has been reported to be associated with mortality, whereas social isolation from friends has not (Shor et al., 2013). However, in the areas of the GEJE, isolation from friends before the disaster is associated with mortality, but not from family members (Aida et al., 2017). Reportedly, social network facilitated cooperation among residents and communities and promoted recovery in the GEJE area (Kawawaki, 2014). The association of social isolation from specific individuals, such as friends and family members, and mortality among survivors living in disaster-affected areas under post-disaster medical and support situations may differ from that in non-disaster areas. Social network outside family, such as friends, might be important in reducing the risk of all-cause mortality in the disaster area. However, no studies have investigated the association between social isolation from family members and friends after a disaster and all-cause mortality in disaster-affected areas.

Thus, we aimed to clarify whether isolation from family members and friends after a disaster is associated with an increased risk of all-cause mortality among survivors living in disaster-affected areas after the GEJE.

## 2 Methods

### 2.1 Study design and population

On March 11, 2011, a massive 9.0-magnitude earthquake (the GEJE) and a tsunami struck Japan's northeastern coast. The Research Project for Prospective Investigation of Health Problems Among Survivors of the GEJE and Tsunami Disaster (RIAS) Study is a population-based longitudinal cohort study launched for the inhabitants of Yamada Town, Otsuchi Town, Rikuzentakata City, and part of Kamaishi City by Iwate Medical University in 2011. The study aimed to investigate the health effects caused by the disaster sustainably and provide proper assistance for the victims and is ongoing. We followed data collection methods described previously in detail (Yokoyama et al., 2014; Tsubota-Utsugi et al., 2018; Kuno et al., 2025). Briefly, among the inhabitants aged ≥18 years as of October 1, 2011, in Yamada Town, Otsuchi Town, and Rikuzentakata City, we sent out notifications of the health survey and questionnaires to 42,286 residents. Among them, 10,057 inhabitants, who provided written informed consent for participation in this study, were included in this study. Of them, we excluded 746 participants for the following reasons: 214 participants did not have complete, or were missing, the Lubben Social Network Scale-6 (LSNS-6) data, and 532 participants did not have complete, or were missing, covariate data. Eventually, 9311 participants (3600 men and 5711 women) were included in this study (Fig. 1).

**Fig. 1:**
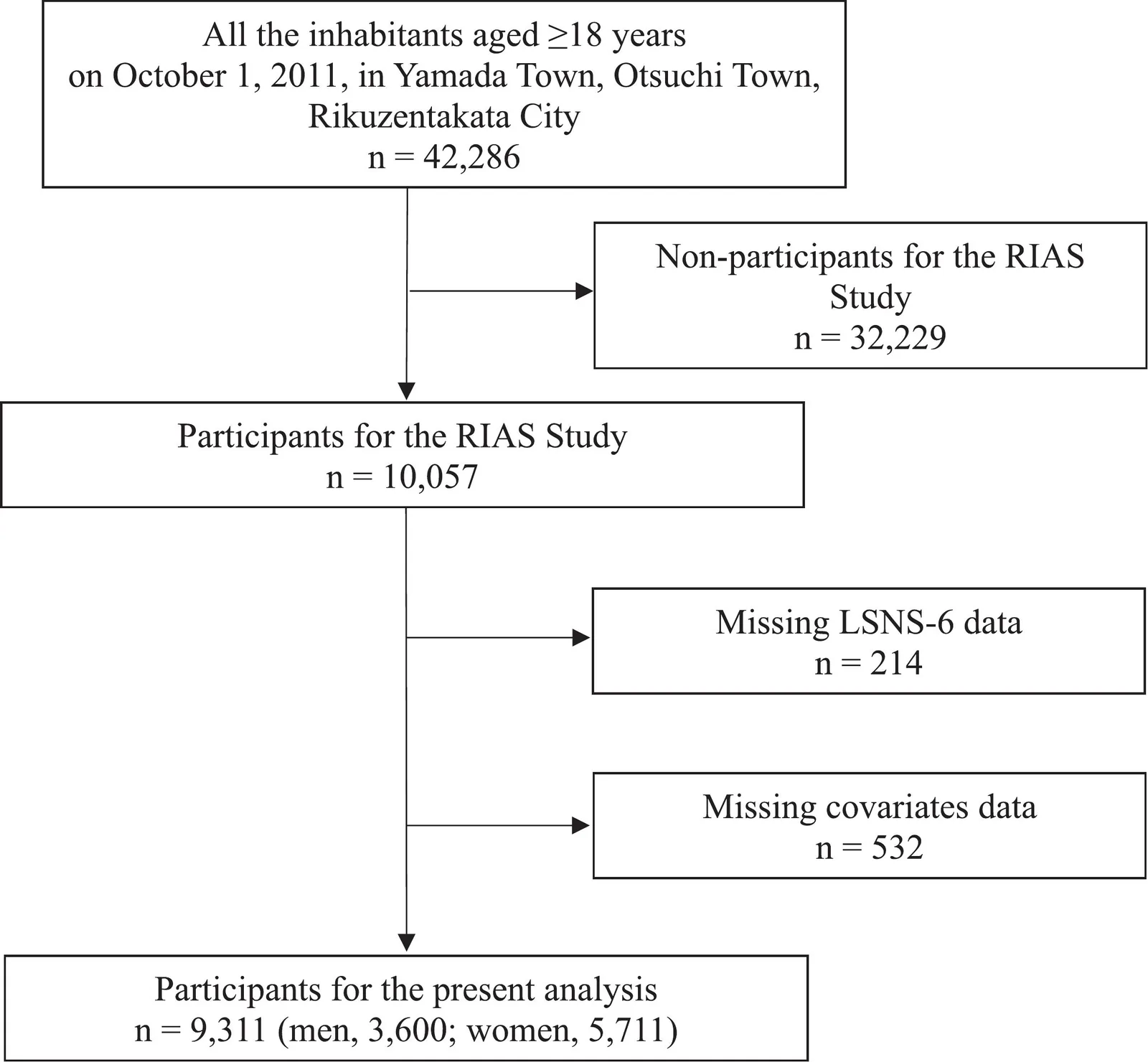
Flow diagram for selection of adults enrolled in the study in Iwate Prefecture, Japan, 2011. The participants were selected from the RIAS study among those with complete data for the questionnaire variables and analysis, resulting in a final analytical sample of 9311 individuals. Abbreviations: RIAS Study, Research Project for Prospective Investigation of Health Problems Among Survivors of the Great East Japan Earthquake and Tsunami Disaster Study.

This study was conducted in accordance with relevant laws and institutional guidelines and was approved by the Institutional Review Board of Iwate Medical University (MH2022–132).

### 2.2 Measures

We assessed social isolation from family only, from friends only, and from both family and friends using the LSNS-6 Family Subscale and the LSNS-6 Friend Subscale (Lubben et al., 2006; Lubben, 1988; Kurimoto et al., 2011). The LSNS-6 consists of three questions that evaluate the number of family members or friends seen or heard from more than once a month, those felt close to such that you can call on them for help, and those felt at ease talking to about private matters. Social isolation from family and friends was defined as a score < 6 based on the answers to the LSNS-6 Family and Friend Subscales, respectively (Lubben et al., 2006).

The primary outcome was death from any cause. The participants were followed up from October 1, 2011, to the occurrence of one of the following censoring events: death, loss to follow-up owing to moving away from the municipalities, or the end of the follow-up period (December 31, 2017). Deaths, dates of death, and cause of death were verified by reviewing the Basic Resident Registers of all municipalities. Residence in the disaster area was confirmed using resident registration cards.

Concurrent with the LSNS-6 assessment, we collected information on age, sex, residential area, smoking and alcohol consumption habits, medication use, dietary habits, physical activity, employment status, economic status, sleep disorders, psychological distress (Sakurai et al., 2011), and relocation owing to the disaster, using a self-administered questionnaire. Smoking habits were assessed by asking the question, “How many cigarettes do you smoke per day?” and classified into two groups: ≥one cigarette/day as current smoking or no smoking. Alcohol drinking habits were assessed by asking the question, “How many times do you drink per week?” and classified into two groups: ≥one time as current alcohol consumption or no drinking. Medications were assessed by asking the question, “Do you have any disease that was treated before the GEJE?” Based on their answers, the participants were categorized into two groups: those who were receiving treatment and those who did not receive treatment. Meal habits were assessed using a question on the frequency of meals per day, and abnormal meal habit was defined as consuming other than three meals per day. Physical activity was assessed using the following three questions: frequency of engaging in physical activity at home and work, frequency of leaving their residence, and length of time spent walking each day. Based on their answers, metabolic equivalents (METs) per week were calculated and categorized into two groups using 22/23 METs-h/week as the cutoff point (Murakami et al., 2013; https://www.mhlw.go.jp/content/001495670.pdf, n.d.). Job status was assessed by asking the questions, “Did you have a job before GEJE” and “Did you change the situation of your job?” and classified into two groups: those who were unemployed before the GEJE and remained unemployed after the GEJE and those who were employed before the GEJE and lost their job after the GEJE as having no job, and others as having a job. Economic status was assessed by asking the question “How do you feel about your current economic situation?” with the following four options: very severe, severe, slightly severe, and normal. Based on their answers, the participants were categorized into two groups: severe (very severe, severe, or slightly severe) and normal. Sleep disorder was assessed using the Athens Insomnia Scale (AIS) and defined as AIS  ≥4 (Okajima et al., 2013). Psychological distress was assessed using the K6 score and defined as K6  ≥5. Relocation owing to the disaster was assessed by a question on the frequency of relocation and categorized into two groups: no relocation and ≥ one time. We also measured blood pressure levels and body weight and height. Systolic and diastolic blood pressure levels were measured twice consecutively in sitting position using an automatic device after urination and a 5-min rest period. The average of the two blood pressure measurements was used for the analysis. Body mass index (BMI) was calculated by measuring height and weight.

### 2.3 Statistical analysis

To evaluate the differences in baseline characteristics between the social isolation and non-social isolation groups, Analysis of Variance and chi-squared tests were conducted for continuous and categorical variables, respectively. The proportional hazards assumptions were verified using Schoenfeld residuals. Kaplan–Meier survival curves were used to obtain the estimates of survival at an average of 5.9 observation years, and the log-rank test was conducted to test for significant differences between survival curves for the social isolation group. Unadjusted and multivariate-adjusted HRs and 95% confidence intervals for all-cause mortality were calculated using Cox proportional hazards models. In the multivariate-adjusted model, age, sex, systolic blood pressure, BMI, current smoking, current alcohol consumption, medical history, other than three meals per day, low physical activity, no job, severe economic status, sleep disorders, psychological distress, and relocation owing to the disaster were treated as covariates. For sensitivity analyses, we performed four additional analyses: (1) cause-specific mortality analyses in which causes of death were classified as cancer (International Classification of Diseases, 10th revision [ICD-10] codes C00–C97), cardiovascular diseases (CVD) (ICD-10 codes I00–I99), or other causes; (2) analyses stratified by age using cutoffs of 65 and 75 years; (3) interactions of social isolation with age (<65 and ≥ 65 years), sex, and relocation owing to the disaster; and (4) an analysis restricted to participants without overall social isolation, assessed using the LSNS-6 (Lubben et al., 2006). *P* values <0.05 were considered statistically significant. Participants with missing values for LSNS-6 and covariates were excluded from the analyses, resulting in a complete-case analysis involving participants with complete data on LSNS-6 and covariates. Complete-case analysis provides unbiased estimates when missingness is independent of the outcome, conditional on the observed variables (missing at random). The proportion of missing data was relatively small (7.5%). The characteristics of participants with and without complete data were compared. All analyses were performed using R version 4.4.2.

## 3 Results

The number (proportion) of participants according to the social isolation group from family only, from friends only, and from both family and friends was 330 (3.5%), 1420 (15.1%), and 416 (4.4%), respectively. Across all measures of social isolation, participants with social isolation from friends had the highest mean age, and there was no significant difference in relocation owing to the disaster between the four groups (Table 1).

**Table 1:** Baseline characteristics of adults enrolled in the study in Iwate Prefecture, Japan, 2011.

LSNS-6 Family Subscale	Non-isolation	Isolation	Non-isolation	Isolation
LSNS-6 Friend Subscale	Non-isolation	Non-isolation	Isolation	Isolation
Number of participants	7145	330	1420	416
Age, years	60.9 (14.6)	54.1 (16.3)	62.6 (13.4)	58.9 (15.3)
*P* value	(ref)	<0.01	<0.01	0.04
Female, %	62.4	62.1	59.2	50.5
*P* value	(ref)	0.99	0.15	<0.01
Systolic blood pressure, mmHg	126.1 (19.0)	122.7 (18.6)	127.3 (18.1)	124.8 (18.8)
*P* value	(ref)	0.01	0.19	0.99
Body mass index, kg/m^2^	23.7 (3.6)	23.8 (4.0)	23.6 (3.4)	23.6 (3.8)
*P* value	(ref)	0.99	0.99	0.99
Current smoking, %	16.6	19.7	13.3	19.2
*P* value	(ref)	0.99	0.01	0.99
Current alcohol consumption, %	32.9	38.8	29.8	34.1
*P* value	(ref)	0.19	0.14	0.99
Diseases currently being treated, %	62.9	60.9	67.7	62.5
*P* value	(ref)	0.99	<0.01	0.99
Meals per day, %	7.1	13.6	6.3	11.3
*P* value	(ref)	<0.01	0.99	0.01
Low physical activity, %	63.5	68.8	71.1	75.7
*P* value	(ref)	0.35	<0.01	<0.01
Having no job, %	59.3	57.0	66.5	65.6
*P* value	(ref)	0.99	<0.01	0.07
Severe economic status, %	49.6	59.4	55.8	63.5
*P* value	(ref)	<0.01	<0.01	<0.01
Sleep disorder, %	49.4	59.4	54.5	61.1
*P* value	(ref)	<0.01	<0.01	<0.01
Psychological distress, %	40.5	46.4	45.0	57.7
*P* value	(ref)	0.24	0.01	<0.01
Relocation owing to the disaster, %	52.1	51.2	51.7	54.6
*P* value	(ref)	0.99	0.99	0.99

Abbreviation: LSNS-6, Lubben Social Network Scale-6.

Continuous variables are expressed as means (standard deviations) and categorical variables as percentages.

*P* values were estimated using the Analysis of Variance for continuous variables and the chi-squared test for categorical variables, with Bonferroni correction for multiple comparisons.

The total observation period was 54,838 person-years, and the mean (standard deviation) observation period was 5.89 (1.02) years with 416 deaths. For the log-rank test, the group with social isolation from friends only had a significantly higher risk of all-cause mortality than the group with neither family nor friend isolation, whereas no significant associations were observed for the groups with social isolation from family only or from both family and friends (Fig. 2). Table 2 shows the HRs (95% confidence interval) for all-cause mortality according to the LSNS-6 Family Subscale and the LSNS-6 Friend Subscale. Compared with participants who were socially isolated from neither family nor friends, those who were socially isolated from friends only had a significantly higher risk of all-cause mortality. Although no significant associations were observed between social isolation and cause-specific mortality in the multivariable analyses, the HRs for cancer and CVD mortality tended to be higher among participants who were socially isolated from friends only and from both family and friends (Table 3). Compared with participants with neither family nor friend isolation, those with social isolation from friends only had a significantly higher risk of all-cause mortality among participants aged ≥65 years, but not among those aged <65 years (Table 4). Regarding age, sex, and relocation owing to the disaster, interactions with social isolation were not observed (Table S1). Among the participants with LSNS-6 score of ≥12, only in LSNS-6 Friend Subscale, social isolation was significantly associated with an increased risk for all-cause mortality (Table S2). Participants excluded due to missing LSNS-6 and covariate data were older than those included in the analysis (Tables S3 and S4).

**Fig. 2:**
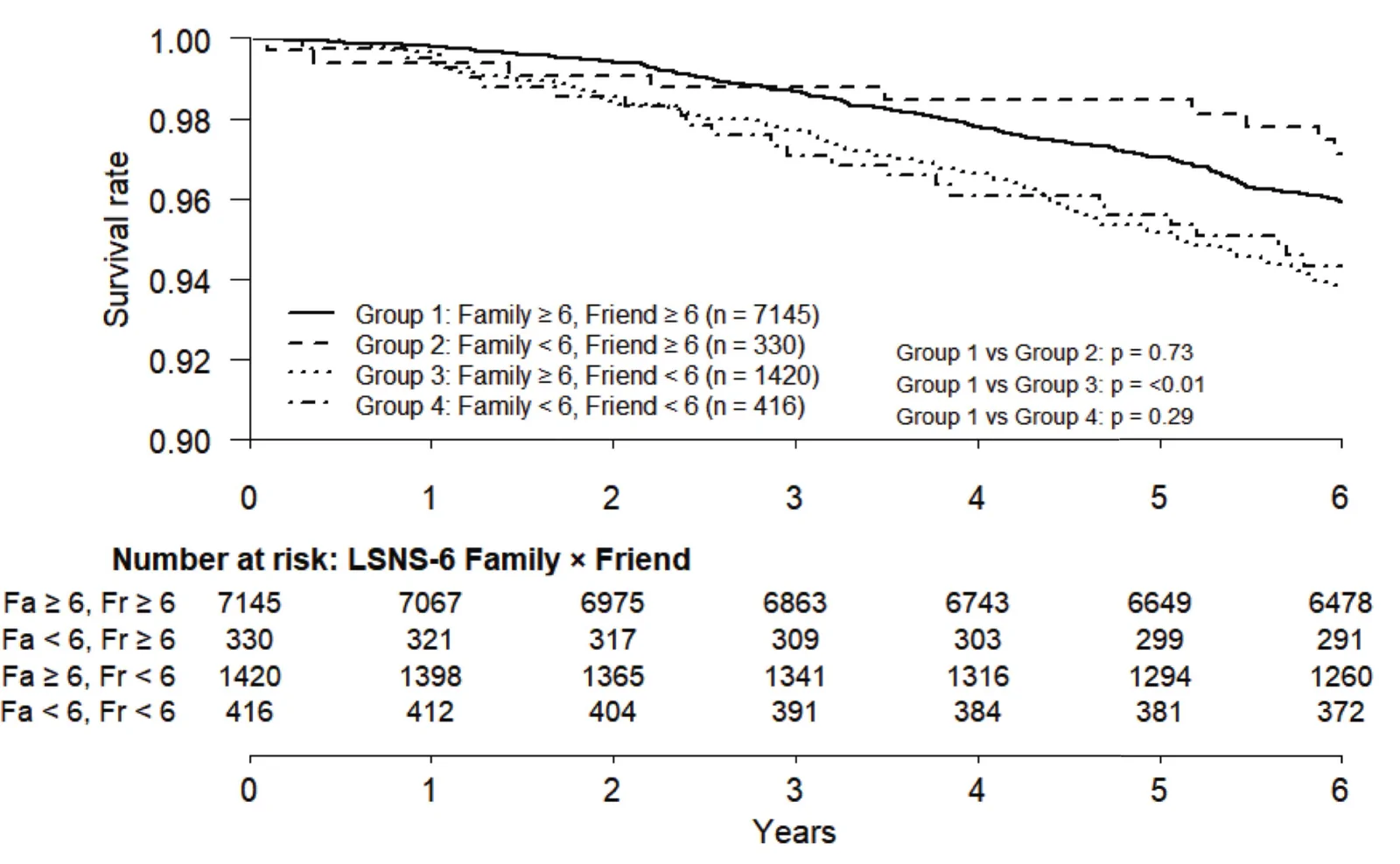
Trend of survival curve and results of the log-rank test among adults enrolled in the study in Iwate Prefecture, Japan, 2011–2017. The horizontal axis represents follow-up time in years, and the vertical axis represents the cumulative survival probability. *P* values were estimated using the log-rank test.

**Table 2:** Number of deaths, mortality rates, and hazard ratios (95% confidence intervals) for all-cause mortality according to social isolation among adults enrolled in the study in Iwate Prefecture, Japan, 2011–2017.

LSNS-6 Family Subscale	Non-isolation	Isolation	Non-isolation	Isolation
LSNS-6 Friend Subscale	Non-isolation	Non-isolation	Isolation	Isolation
Number of participants	7145	330	1420	416
Observation (person-years)	42,227	1910	8273	2430
Number of deaths	295	9	88	24
Mortality rate (/1000 person-years)	7.0	4.7	10.6	9.9
Unadjusted HR (95% CI)	1.00 (ref)	0.66 (0.35,1.31)	1.53 (1.20,1.94)	1.41 (0.94,2.15)
Multivariate-adjusted HR (95% CI)	1.00 (ref)	0.93 (0.48,1.81)	1.28 (1.01,1.63)	1.22 (0.80,1.86)

Abbreviations: HR, hazard ratio; CI, confidence interval.

Multivariate-adjusted HRs (95% CIs) were calculated using Cox's proportional hazards model by adjusting for age, sex, systolic blood pressure, body mass index, current smoking habits, current alcohol consumption habits, medical history, other than three meals per day, low physical activity, no job, severe economic status, sleep disorder, psychological distress, and relocation owing to the disaster.

**Table 3:** Number of deaths, mortality rates, and hazard ratios (95% confidence intervals) for cause-specific mortality according to social isolation among adults enrolled in the study in Iwate Prefecture, Japan, 2011–2017.

LSNS-6 Family Subscale	Non-isolation	Isolation	Non-isolation	Isolation
LSNS-6 Friend Subscale	Non-isolation	Non-isolation	Isolation	Isolation
*Cancer*				
Number of deaths	140	3	38	14
Mortality rate (/1000 person-years)	3.3	1.6	4.6	5.8
Unadjusted HR (95% CI)	1.00 (ref)	0.47 (0.15,1.49)	1.39 (0.97,1.99)	1.74 (1.01,3.09)
Multivariate-adjusted HR (95% CI)	1.00 (ref)	0.65 (0.21,2.04)	1.24 (0.87,1.79)	1.53 (0.87,2.67)
*CVD*				
Number of deaths	74	1	25	6
Mortality rate (/1000 person-years)	1.8	0.5	3.0	2.5
Unadjusted HR (95% CI)	1.00 (ref)	0.30 (0.04,2.15)	1.73 (1.10,2.72)	1.41 (0.61,3.24)
Multivariate-adjusted HR (95% CI)	1.00 (ref)	0.43 (0.06,3.11)	1.42 (0.90,2.26)	1.31 (0.56,3.05)
*Other than cancer and CVD*				
Number of deaths	81	5	25	4
Mortality rate (/1000 person-years)	1.91	2.6	3.0	1.64
Unadjusted HR (95% CI)	1.00 (ref)	1.37 (0.56,3.38)	1.58 (1.01,2.47)	0.86 (0.32,2.35)
Multivariate-adjusted HR (95% CI)	1.00 (ref)	1.83 (0.74,4.55)	1.21 (0.77,1.91)	0.71 (0.26,1.95)

Abbreviations: HR, hazard ratio; CI, confidence interval.

Multivariate-adjusted HRs (95% CIs) were calculated using Cox's proportional hazards model by adjusting for age, sex, systolic blood pressure, body mass index, current smoking habits, current alcohol consumption habits, medical history, other than three meals per day, low physical activity, no job, severe economic status, sleep disorder, psychological distress, and relocation owing to the disaster.

**Table 4:** Number of deaths, mortality rates, and hazard ratios (95% confidence intervals) for all-cause mortality according to social isolation among adults aged ≥65 years and < 65 years in Iwate Prefecture, Japan, 2011–2017.

LSNS-6 Family Subscale	Non-isolation	Isolation	Non-Isolation	Isolation
LSNS-6 Friend Subscale	Non-isolation	Non-isolation	Isolation	Isolation
*aged ≥ 65 years*				
Number of participants	3413	99	671	166
Observation (person-years)	20,088	557	3820	944
Number of deaths	249	8	79	19
Mortality rate (/1000 person-years)	12.4	14.4	20.7	20.1
Unadjusted HR (95% CI)	1.00 (ref)	1.16 (0.57,2.35)	1.68 (1.30,2.16)	1.64 (1.03,2.61)
Multivariate-adjusted HR (95% CI)	1.00 (ref)	1.01 (0.50,2.06)	1.31 (1.01,1.70)	1.13 (0.70,1.81)
*aged < 65 years*				
Number of participants	3732	231	749	250
Observation (person-years)	22,138	1353	4453	1486
Number of deaths	46	1	9	5
Mortality rate (/1000 person-years)	2.1	0.7	2.0	3.4
Unadjusted HR (95% CI)	1.00 (ref)	0.36 (0.05,2.59)	0.97 (0.48,1.99)	1.62 (0.64,4.08)
Multivariate-adjusted HR (95% CI)	1.00 (ref)	0.48 (0.07,3.53)	0.89 (0.43,1.82)	1.53 (0.60,3.90)

Abbreviations: HR, hazard ratio; CI, confidence interval.

Multivariate-adjusted HRs (95% CIs) were calculated using Cox's proportional hazards model by adjusting for age, sex, systolic blood pressure, body mass index, current smoking habits, current alcohol consumption habits, medical history, other than three meals per day, low physical activity, no job, severe economic status, sleep disorder, psychological distress, and relocation owing to the disaster.

## 4 Discussion

Our findings demonstrate that compared with participants with neither family nor friend isolation, those with social isolation from friends only had a significantly higher risk of all-cause mortality among survivors living in disaster-affected areas after the GEJE. This suggests that social isolation from friends increases the risk of premature death after a large-scale disaster.

Our findings are consistent with those of a previous study, which used the social isolation index, assessed before a disaster and reported that social isolation from friends contributed to an increase in all-cause mortality (Aida et al., 2017). Social network effects facilitating cooperation among residents and communities and promoting recovery (Kawawaki, 2014) might lead to these results. Social network outside the family, such as friends, might be important in reducing the risk of all-cause mortality in the disaster area.

Although not statistically significant, HRs for cancer and CVD mortality tended to be higher among socially isolated from friends only and from both family and friends, suggesting that these causes may have contributed to their increased all-cause mortality. Previous studies have linked social isolation to cancer and CVD mortality (Wang et al., 2023), but evidence specific to friend isolation is limited to breast cancer (Kroenke et al., 2006). The small number of cause-specific deaths may have limited statistical power. Further studies should examine associations between specific components of social isolation and cause-specific mortality.

In age-stratified analyses, social isolation from friends was associated with increased all-cause mortality only among participants aged ≥65 years. Previous studies have linked social isolation to mortality in older adults (Nakou et al., 2025), and social connections with friends to 10-year survival among community-dwelling adults aged ≥70 years (Giles et al., 2005). To our knowledge, no study has examined friend isolation and mortality using age-stratified analyses. Our findings suggest that addressing friend isolation may be particularly important for reducing mortality among older adults, even in disaster-affected areas. No significant association was observed among participants aged <65 years, possibly due to limited statistical power from fewer deaths. Further studies should clarify this association in younger individuals.

Among participants with an LSNS-6 score ≥ 12, social isolation from friends remained associated with increased all-cause mortality, suggesting that the overall LSNS-6 score may overlook at-risk individuals isolated from friends. The comprehensive index including LSNS-6 could reduce the effect of the factor with the largest effect. Another study assessed isolation and mortality using a comprehensive index (Wang et al., 2023). Assessing this theme, considering the content of social isolation, is the next challenge.

Unlike family relationships, interactions with friends may more strongly reflect voluntary social engagement and connections outside the household, which may provide distinct emotional and psychosocial benefits. In the area affected by the Great East Japan Earthquake, a previous study showed that increased neighborhood ties after the disaster were associated with a reduced risk of depressive symptoms (Sasaki et al., 2020) and another study reported that depression was associated with an increased risk of all-cause mortality during 3.3 years of follow-up among older disaster survivors (Li et al., 2019). These findings suggest that depression may be one potential pathway linking social isolation from friends to all-cause mortality. In this study, social isolation from friends remained significantly associated with all-cause mortality even after adjustment for psychological distress assessed using the K6. However, the previous study assessed depressive symptoms using the Geriatric Depression Scale (Sasaki et al., 2020; Li et al., 2019) whereas the K6 used in our study measures psychological distress and may not have fully captured depressive symptoms. Therefore, further studies are needed to clarify the mechanisms underlying the association between social isolation from friends and increased mortality risk after a disaster.

The association between friend isolation and post-diagnosis outcomes may vary across countries and populations. Social support was not associated with hypertension control or medication adherence among Nepalese patients (Bhattarai et al., 2024), while friend caregiving support was negatively associated with optimal medication adherence among Chinese patients receiving hypertension management services (Shen et al., 2022).

Social isolation from family members was not associated with all-cause mortality in this study. The isolation from family members was associated with mortality in the non-disaster area (Shor et al., 2013), which was not the same as that observed in our study. This may be because the victims' families in the disaster area were also affected by the disaster and could not help each other.

This study has some limitations. First, social isolation was assessed only once. Thus, we could not determine whether social isolation was caused by the disaster, nor could we consider the change in social isolation after the disaster. Second, there might have been residual confounding factors that could not be considered. Third, self-selection bias may have occurred because this study included only those participants who had undergone a health checkup. Contact with friends is associated with the uptake of health checkups by welfare recipients (Hayashi, 2025). This implies that the results of this survey, which excluded non-health checkup recipients, are conservative, and the true effect may be greater than that suggested by the results. Fourth, this study was conducted in a disaster area affected by a large-scale earthquake and tsunami. Attention should be paid when generalizing our results to other disasters or to areas outside the disaster-affected areas. Fifth, participants with missing covariate data differed from those included in the analysis, which may limit the generalizability of our findings. Further research is needed on the characteristics of participants with missing covariate data and on methods to prevent data loss.

## 5 Conclusions

In conclusion, social isolation from friends is significantly associated with an increased risk of all-cause mortality among survivors living in disaster-affected areas after the GEJE. After a large-scale natural disaster, a policy that focuses more on preventive medical support and preservation of social networks, enabling us to communicate with friends and neighbors, is required, and we should consider which interventions can implement these initiatives and prevent premature deaths after the disaster.

## Dual authorship

This manuscript is original, has not been published previously, and is not under consideration for publication elsewhere. No part of this work has been submitted to another journal.

## CRediT authorship contribution statement

**Satoru Kanda:** Writing – review & editing, Writing – original draft, Visualization, Validation, Supervision, Software, Resources, Project administration, Methodology, Investigation, Funding acquisition, Formal analysis, Data curation, Conceptualization. **Eri Takusari:** Writing – review & editing, Writing – original draft, Visualization, Validation, Supervision, Software, Resources, Project administration, Methodology, Investigation, Funding acquisition, Formal analysis, Data curation, Conceptualization. **Haruki Shimoda:** Writing – review & editing, Writing – original draft, Validation, Supervision, Resources, Project administration, Methodology, Investigation, Funding acquisition, Data curation, Conceptualization. **Kiyomi Sakata:** Writing – review & editing, Writing – original draft, Validation, Supervision, Resources, Project administration, Methodology, Investigation, Funding acquisition, Data curation, Conceptualization. **Kozo Tanno:** Writing – review & editing, Writing – original draft, Visualization, Validation, Supervision, Software, Resources, Project administration, Methodology, Investigation, Funding acquisition, Formal analysis, Data curation, Conceptualization.

## Disclaimer

None.

## Ethics declaration

### Informed consent and patient details

Written informed consent to take part in the study and to publish the article has been obtained from all participants or their legal representatives. The privacy rights of participants have been observed.

### Studies in human

This study was performed in compliance with relevant laws, regulatory frameworks and guidelines where the research took place. This study was conducted in accordance with Declaration of Helsinki. This study was approved by the Institutional Review Board of Iwate Medical University. (Approval No. MH2022-132).

## Declaration of competing interest

The authors declare that they have no known competing financial interests or personal relationships that could have appeared to influence the work reported in this paper.

## Data Availability

As our data are sensitive and are maintained based on agreement with the study participants, they are not available to other researchers to reproduce the results or replicate the procedure. However, our data have not been shared.

## Appendix A Supplementary data

Supplementary Table S1 Hazard ratios (95% confidence intervals) for all-cause mortality according to interactions of age, sex, and relocation with social isolation among adults enrolled in the study in Iwate Prefecture, Japan, 2011–2017.

Supplementary Table S2 Number of deaths, mortality rates, and hazard ratios (95% confidence intervals) for all-cause mortality according to the LSNS-6 Family Subscale and LSNS-6 Friend Subscale among only social non-isolation participants assessed using the LSNS-6 among adults without overall social isolation in Iwate Prefecture, Japan, 2011–2017.

Supplementary Table S3 Characteristics of adults with complete or missing social network data in Iwate Prefecture, Japan, 2011.

Supplementary Table S4 Characteristics of adults with complete or missing covariate data in Iwate Prefecture, Japan, 2011.
