# Supplementary material for: Association between types of social isolation and all-cause mortality among the survivors in the disaster-affected area of the great East Japan earthquake: The RIAS study
Source: Prev Med Rep. 2026 Sep 30;71:103654. doi: 10.1016/j.pmedr.2026.103654 (PMC13659327; doi:10.1016/j.pmedr.2026.103654)
Supplement: Supplementary file 1 [file mmc1.docx]

Table S1. Hazard ratios (95% confidence intervals) for all-cause mortality according to interactions of age, sex, and relocation with social isolation among adults enrolled in the study in Iwate Prefecture, Japan, 2011–2017.

| LSNS-6 Family Subscale | Non-isolation | isolation | Non-Isolation | Isolation |
| --- | --- | --- | --- | --- |
| LSNS-6 Friend Subscale | Non-isolation | Non-Isolation | isolation | Isolation |
| *Age interactions^a^ (95% CI)* | 1.00 (ref) | 1.03 (0.96,1.12) | 1.01 (0.99,1.04) | 0.98 (0.94,1.01) |
| *Sex interactions^b^ (95% CI)* | 1.00 (ref) | 0.70 (0.17,2.84) | 0.97 (0.58,1.62) | 1.91 (0.81,4.54) |
| *Relocation interactions^c^ (95% CI)* | 1.00 (ref) | 0.21 (0.03,1.68) | 1.03 (0.64,1.67) | 1.50 (0.65,3.48) |

Abbreviations: HR, hazard ratio; CI, confidence interval.

Multivariate-adjusted HRs (95% CIs) were calculated using Cox’s proportional hazards model by adjusting for age, sex, systolic blood pressure, body mass index, current smoking habits, current alcohol consumption habits, medical history, other than three meals per day, low physical activity, no job, severe economic status, sleep disorder, psychological distress, and relocation owing to the disaster

^a^In addition to the covariates listed above, interactions of age with social isolation were included as covariates. For calculating interactions with age, participants were categorized into two groups by age: those aged  ≥ 65 years and those aged < 65 years.

^b^In addition to the covariates listed above, interactions of sex with social isolation were included as covariates.

^c^In addition to the covariates listed above, interactions of relocation owing to the disaster with social isolation were included as covariates.
