# Supplementary material for: Association between types of social isolation and all-cause mortality among the survivors in the disaster-affected area of the great East Japan earthquake: The RIAS study
Source: Prev Med Rep. 2026 Sep 30;71:103654. doi: 10.1016/j.pmedr.2026.103654 (PMC13659327; doi:10.1016/j.pmedr.2026.103654)
Supplement: Supplementary file 2 [file mmc2.docx]

Table S2. Number of deaths, mortality rates, and hazard ratios (95% confidence intervals) for all-cause mortality according to the LSNS-6 Family Subscale and LSNS-6 Friend Subscale among only social non-isolation participants assessed using the LSNS-6 among adults without overall social isolation in Iwate Prefecture, Japan, 2011–2017.

|  | LSNS-6 Family Subscale | | LSNS-6 Friend Subscale | |
| --- | --- | --- | --- | --- |
|  | Non-isolation | Isolation | Non-isolation | Isolation |
| Number of participants | 8,104 | 195 | 7,340 | 959 |
|  |  |  |  |  |
| Observation  (person-years) | 47,804 | 1,124 | 43,350 | 5,577 |
| Number of deaths | 358 | 7 | 302 | 63 |
| Mortality rate  (/1,000 person-years) | 7.5 | 6.2 | 7.0 | 11.3 |
|  |  |  |  |  |
| Unadjusted HR  (95% CI) | 1.00 (ref) | 0.84  (0.40,1.76) | 1.00 (ref) | 1.62  (1.24,2.13) |
|  |  |  |  |  |
| Multivariate-adjusted HR  (95% CI) | 1.00 (ref) | 1.14  (0.54,2.45) | 1.00 (ref) | 1.34  (1.02,1.76) |

Abbreviations: HR, hazard ratio; CI, confidence interval.

Multivariate-adjusted HRs (95% CIs) were calculated using Cox’s proportional hazards model by adjusting for age, sex, systolic blood pressure, body mass index, current smoking habits, current alcohol consumption habits, medical history, other than three meals per day, low physical activity, no job, severe economic status, sleep disorder, psychological distress, and relocation owing to the disaster
