# Supplementary material for: Association between types of social isolation and all-cause mortality among the survivors in the disaster-affected area of the great East Japan earthquake: The RIAS study
Source: Prev Med Rep. 2026 Sep 30;71:103654. doi: 10.1016/j.pmedr.2026.103654 (PMC13659327; doi:10.1016/j.pmedr.2026.103654)
Supplement: Supplementary file 3 [file mmc3.docx]

Table S3. Characteristics of adults with complete or missing social network data in Iwate Prefecture, Japan, 2011.

|  | Complete LSNS-6 data (n = 9843) | Missing LSNS-6 data (n = 214) |
| --- | --- | --- |
| Age, years | 60.9 | 65.1 |
| Female, % | 61.0 | 62.6 |
| Systolic blood pressure, mmHg | 126.1 | 125.2 |
| Body mass index, kg/m^2^ | 23.7 | 23.5 |
| Current smoking, % | 16.4 | 14.5 |
| Current alcohol consumption, % | 32.9 | 29.9 |
| Diseases currently being treated, % | 63.6 | 71.0 |
| Meals per day, % | 7.4 | 6.1 |
| Low physical activity, % | 65.4 | 77.1 |
| Having no job, % | 60.6 | 68.1 |
| Severe economic status, % | 51.7 | 54.7 |
| Sleep disorder, % | 51.0 | 48.6 |
| Psychological distress, % | 42.1 | 40.2 |
| Relocation owing to the disaster, % | 51.8 | 59.8 |

Abbreviation: LSNS-6, Lubben Social Network Scale-6.

Continuous variables are expressed as means (standard deviations) and categorical variables as percentages.
