# Supplementary material for: Association between types of social isolation and all-cause mortality among the survivors in the disaster-affected area of the great East Japan earthquake: The RIAS study
Source: Prev Med Rep. 2026 Sep 30;71:103654. doi: 10.1016/j.pmedr.2026.103654 (PMC13659327; doi:10.1016/j.pmedr.2026.103654)
Supplement: Supplementary file 4 [file mmc4.docx]

Table S4. Characteristics of adults with complete or missing covariate data in Iwate Prefecture, Japan, 2011.

|  | Complete covariate data (n = 9311) | Missing covariate data (n = 532) |
| --- | --- | --- |
| Age, years | 60.9 | 62.0 |
| Female, % | 61.3 | 54.3 |
| Systolic blood pressure, mmHg | 126.1 | 126.9 |
| Body mass index, kg/m^2^ | 23.7 | 24.0 |
| Current smoking, % | 16.3 | 18.4 |
| Current alcohol consumption, % | 32.7 | 36.3 |
| Diseases currently being treated, % | 63.6 | 64.7 |
| Meals per day, % | 7.4 | 7.1 |
| Low physical activity, % | 65.4 | 65.0 |
| Having no job, % | 60.6 | 61.5 |
| Severe economic status, % | 51.5 | 54.1 |
| Sleep disorder, % | 51.0 | 49.8 |
| Psychological distress, % | 42.2 | 40.0 |
| Relocation owing to the disaster, % | 52.1 | 45.9 |

Continuous variables are expressed as means (standard deviations) and categorical variables as percentages.
